# 4481 Better Together Harrisburg: Community-Driven Research Day

**DOI:** 10.1017/cts.2020.260

**Published:** 2020-07-29

**Authors:** Andrea Murray, Martha Wadsworth, Jennifer Kraschnewski, Kathleen Best, Carmen Henry-Harris

**Affiliations:** 1 Penn State Clinical and Translational Science Institute

## Abstract

OBJECTIVES/GOALS: The overall goal of the Community-Engaged Research Core, supported by the Penn State Clinical and Translational Science Institute, is to invest in opportunities that promote collaboration between researchers and communities. Research in which community members are participating in the research process will more likely lead to reducing health disparities when compared to more traditional approaches. This abstract describes a community research day that brought researchers and community-based organizational leaders together to discuss critical areas of research. We aim to highlight a successful approach for how to work with a community, particularly one that has been distrustful of research, to facilitate and support collaborations between academic researchers and community-based organizational leaders (CBOs). Community-based organizational leaders are often the most knowledgeable individuals when it comes to identifying and discerning the needs and research priorities of their communities and they are generally in the best positions to help build greater trust between academic researchers and communities. METHODS/STUDY POPULATION: A Community Research Day Steering Committee was formed in the spring of 2018 and consisted of 10 community-based organizational leaders from Harrisburg, Pennsylvania, two Penn State University staff, and one Penn State University faculty member. The Steering Committee’s purpose was to design, plan, and execute an event (Better Together: Community Driven Awareness) in which community-organizational leaders and faculty researchers came together to discuss possible research collaborations to improve community health. The Steering Committee participated in bi-monthly planning meetings leading up to the event, Better Together: Community-Driven Awareness. During these planning meetings, members determined that mental health and nutrition were two critical areas deserving of more attention from research within their geographical community. Organizations were asked to identify sub-categories within mental health and nutrition that they saw as most relevant to their communities. The sub-categories that they selected became the theme topics for round table discussions at the main event. This information was also used to determine which academic researchers to invite to the event, based on scientific expertise. In addition to selecting these topics for table discussions, the Steering Committee provided advice on the agenda and program materials. The agenda for Better Together: Community-Driven Awareness featured a presentation from a successful collaboration between a faculty member and a community-based organization whose project was centered around suicide prevention in the school system. After the presentation, researchers and CBOs sat at round tables for facilitated discussions about their table’s theme. The facilitated discussions fostered new relationships and led to collaborations outside of the event. Following the round-table discussions, there was a presentation about funding and next steps. Lastly, feedback forms were given to each attendee to assess their experience of the event and to better understand what to improve upon for the future. RESULTS/ANTICIPATED RESULTS: Following the Community-Driven Awareness event, the Community-Engaged Research Core at Penn State released a call for proposals for planning grants to be awarded to faculty/community-based organization teams. These grants were intended to build capacity for externally-funded research that seeks to address important community-identified research questions. The internal grants support meetings to discuss mutual interests, develop research questions, identify leaders, conduct literature reviews, and collect pilot data. A team must have included, at a minimum, one Penn State faculty researcher and one community-based organizational leader as co-principal investigators. In the proposal, the team was asked to describe its preliminary research question, the work to be accomplished during the planning period, anticipated outcome(s) and deliverables, and preliminary ideas for seeking future external funding. A two-page narrative briefly described how the team members’ expertise/experience/constituencies would address the specified research question. In addition, the team provided a budget and budget justification. Planning grants ranged from $500-$5,000. Funds were allocated for a 6-12 month period. After the call was sent out, seven proposals were submitted and three were selected for external funding. Proposal topics included: * Exploring the Mechanism of Engagement in HIV Testing, Prevention, and Care Among African American and Hispanic/Latino Men who Have Sex with Men * Educator Translation of a Universal Social-Emotional Learning Program in School Practice * Growing Nutritious Communities: Gardening to increase access to and knowledge about fresh fruits and vegetables among residents in South Harrisburg, Hall Manor community. DISCUSSION/SIGNIFICANCE OF IMPACT: There are several academic institutions that have implemented similar events whose goal is to bring together academic researchers and community-based organizational leaders. To our knowledge, this is one of a few examples of an event that was developed from the ground up by a committee comprised mostly of community organization leaders. The community leaders guided the decisions made in all phases of the event design from determining the research themes to providing input on program materials. Additionally, our Steering Committee garnered the interest and attendance from over 20 community participating organizations, which attests to their commitment and dedication to seeing this event through from beginning to end. The feedback received from the event was overwhelmingly positive. Both academic researchers and community-based organizational leaders expressed their appreciation for an event that brought both parties together in a space where they felt comfortable to share ideas and knowledge. When asked how we could improve this event in the future, most attendees shared that they wanted more time and more opportunities to connect. One limitation of the event noted by attendees was that attendees were not able to sign up for the round table discussions themselves but were placed strategically at them by our Steering Committee. Therefore, at our next event, attendees will be able to select their tables and determine which themed topic they prefer to participate in. Lastly, we are considering how to best summarize the ideas that are generated from these round table discussions in a way that can be shared with the larger group and in a way that might foster collaborations outside of the event.

